# The diagnostic accuracy of digital, infrared and mercury-in-glass thermometers in measuring body temperature: a systematic review and network meta-analysis

**DOI:** 10.1007/s11739-020-02556-0

**Published:** 2020-11-25

**Authors:** Valentina Pecoraro, Davide Petri, Giorgio Costantino, Alessandro Squizzato, Lorenzo Moja, Gianni Virgili, Ersilia Lucenteforte

**Affiliations:** 1 Department of Laboratory Medicine and Pathology, Ospedale Civile Sant’Agostino Estense, AUSL Modena, Modena, Italy; 2 Department of Clinical and Experimental Medicine, University of Pisa, Via Roma, 10, 56126 Pisa, Italy; 3 IRCCS Fondazione Ca’ Granda, Ospedale Maggiore Policlinico, UOC Pronto Soccorso e Medicina D’Urgenza, Università Degli Studi di Milano, Milan, Italy; 4 Department of Medicine and Surgery, University of Insubria, Como, Italy; 5 Department of Biomedical Sciences for Health, University of Milan, Milan, Italy; 6 Department of Neurosciences, Psychology, Drug Research and Child Health (NEUROFARBA), AOU Careggi, Florence, Italy

**Keywords:** Body temperature, Diagnostic tests, Fever, Systematic review, Thermometers

## Abstract

Not much is known about how accurate and reproducible different thermometers are at diagnosing patients with suspected fever. The study aims at evaluating which peripheral thermometers are more accurate and reproducible. We searched Medline, Embase, Scopus, WOS, CENTRAL, and Cinahl to perform: (1) diagnostic accuracy meta-analysis (MA) using rectal mercury-in-glass or digital thermometry as reference, and bivariate models for pooling; (2) network MA to estimate differences in mean temperature between devices; (3) Bland–Altman method to estimate 95% coefficient of reproducibility. PROSPERO registration: CRD42020174996. We included 46 studies enrolling more than 12,000 patients. Using 38 °C (100.4 ℉) as cut-off temperature, temporal infrared thermometry had a sensitivity of 0.76 (95% confidence interval, 0.65, 0.84; low certainty) and specificity of 0.96 (0.92, 0.98; moderate certainty); tympanic infrared thermometry had a sensitivity of 0.77 (0.60,  0.88; low certainty) and specificity of 0.98 (0.95, 0.99; moderate certainty). For all the other index devices, it was not possible to pool the estimates. Compared to the rectal mercury-in-glass thermometer, mean temperature differences were not statistically different from zero for temporal or tympanic infrared thermometry; the median coefficient of reproducibility ranged between 0.53 °C [0.95 ℉] for infrared temporal and 1.2 °C [2.16 ℉] for axillary digital thermometry. Several peripheral thermometers proved specific, but not sensitive for diagnosing fever with rectal thermometry as a reference standard, meaning that finding a temperature below 38 °C does not rule out fever. Fixed differences between temperatures together with random error means facing differences between measurements in the order of 2 °C [4.5 ℉]. This study informs practitioners of the limitations associated with different thermometers; peripheral ones are specific but not sensitive.

**Electronic supplementary material:**

The online version of this article (10.1007/s11739-020-02556-0) contains supplementary material, which is available to authorized users.

## Introduction

Body temperature is a vital parameter. Fever (or pyrexia) is the temporary pathological state that involves an alteration of the hypothalamic thermoregulation system and a consequent elevation of body temperature above the value considered normal. Many diseases begin with increased body temperature, determining a febrile state. Although there is no single agreed threshold for diagnosing fever, a value above the interval between 37.7 °C and 38.3 °C is usually considered a febrile response [1]. Measurement of body temperature depends on the selection of the anatomical area, with marked differences between the body core temperature and the surface temperature [2]. Another important source of variability is that body temperature changes during the course of the day and depends on a person’s activity.

Fever originates from infections (e.g. viral, bacterial) and from non-infectious conditions (e.g. inflammation, malignancies, autoimmune disease, drug adverse events), and in some cases, its aetiology is of unknown origin**.** Fever is also a common symptom of COVID-19, typically appearing 2–14 days after exposure. Therefore, clinical electronic thermometers are an important screening and diagnostic tool to assist in the identification of those individuals who may be infected with COVID-19 [3].

Determination of body temperature is a key clinical action in the management of patients: the presence of fever affects the decision of clinicians, patients, and caregivers, impacting diagnosis, investigations, and therapies (e.g. antibiotic administration). So, accurate measurement of temperature is essential, and thermometers should accurately measure body temperature oscillations.

The US Food and Drug Administration acknowledges the fact that non-contact temperature assessment devices are not effective if used as the only means of detecting a COVID-19 infection. This failure is not only related to the absence of fever in some affected patients, but also because devices fail to identify elevated temperature, or misread normal temperature as elevated. Moreover, failure to follow the manufacturer’s instructions for use, such as for set-up, operation, and training, is also reported as a limitation of non-contact thermometer use [4].

There are several types of medical thermometers. Mercury-in-glass thermometers were the standard reference method for decades [5–7], until the late 2000s when they were banned from the market due to the environmental toxicity of mercury [8]. Alternative thermometers have come into use, such as digital tympanic or axillary, infrared skin scan, temporal artery thermometers, and non-contact infrared thermometers.

Despite the central role of thermometers in clinical practice, our knowledge of the relative performance of alternative thermometers, including differences in measured temperature, is limited. Consequently, it is necessary to understand the characteristics and diagnostic accuracy of different thermometers, appreciating their limitations as tools that guide patient management. This is particularly important given the triage role of fever measurement in several clinical settings, particularly in emergency care settings, with the aim of sending potential COVID-19 patients to appropriate care pathways.

We systematically reviewed studies comparing the accuracy of digital, infrared and mercury-in-glass thermometers, estimating body temperature on different anatomical sites, both in adults and children.

## Methods

We performed a systematic review and meta-analysis according to the recommendations indicated in the Cochrane Handbook for Diagnostic Test Accuracy Reviews [9]. Moreover, we used NMA methods to compare multiple diagnostic tests and body sites in one simultaneous analysis. For this purpose, we extracted between-test differences and used them as a continuous variable to fit standard NMA techniques.

The reporting was in accordance with the Preferred Reporting Items for Systematic reviews and Meta-Analyses of Diagnostic Test Accuracy Studies (PRISMA-DTA) criteria [10].

This systematic review has been registered on PROSPERO 2020 (CRD42020174996).

### Search strategy

We performed a systematic search up to March 2020 on six electronic databases: Medline, Embase, Web of Science (WOS), Scopus, The Cochrane Central Register of Controlled Trials (CENTRAL), and Cinhal, to identify all possible eligible studies. These databases were searched using the following search keywords: “sensitivity”, “specificity”, “body temperature”, “thermometer”. The search strategy was first developed for Medline and then adapted to all other databases. Finally, we checked the reference list of all selected studies.

### Patients

We included adult and child patients screened for fever in emergency and hospital in-patient departments.

### Index and reference standard thermometer categories

The thermometer type was classified as mercury-in-glass, infrared or other digital devices [11]. The body sites considered were grouped as peripheral (i.e. tympanic, temporal artery, axillary, and oral) or central (i.e. rectal, pulmonary artery, urinary bladder, and oesophageal sites) [12, 13]. For diagnostic accuracy analyses, we assumed that mercury-in-glass or digital thermometry at the rectal site was the reference standard. Because of the limited number of studies, we conducted separate analyses for body site and thermometer type. For network meta-analyses of mean differences, we considered rectal mercury-in-glass as the reference category.

### Outcomes

The primary outcome was the diagnostic accuracy of digital, infrared, and mercury-in-glass thermometers defined as the number of true positives (TP), false positives (FP), false negatives (FN), and true negatives (TN) reported in each study. When these data were not available, they were calculated from sensitivity and specificity data. We also evaluated the mean difference in temperature determined using different types of thermometers (fixed bias) and reported as 1.96 times the standard deviation (SD) of these differences (random error), which are the two components of the 95% coefficient of reproducibility [14].

### Study selection

We included studies which respected the following eligibility criteria: (i) randomized clinical trial, observational cohort or cross-sectional study; (ii) enrolled adults or children accessing an emergency department (ED), enrolled adult or child patients hospitalized in hospital or in neonatal departments; (iii) studies that considered rectal or axillary temperature as the reference standard, measured with mercury-in-glass or digital thermometers; (iv) body temperature measured by clinicians or nurses; (v) studies that provided sensitivity and specificity data and temperature measured with each thermometer used; (vi) published in English, Italian, Spanish, or French. We excluded surgical patients, studies where body temperature was measured by mothers or using only one type of thermometer. After removing duplicates, two independent authors screened titles and abstracts and identified all potentially eligible studies. The full text of selected citations was then reviewed according to the inclusion criteria.

### Data extraction

One author used a standardized data extraction form to collect relevant publication details regarding study methods and results, and the second author checked the data. The authors collected data about: (i) study characteristics (i.e. authors, year of publication, title, reference, study design, eligibility criteria and setting); (ii) patient characteristics (i.e. age, number of enrolled patients, and site of measurement of body temperature); (iii) detailed information about the index test (i.e. any other type of body thermometer) and reference standard (i.e. mercury-in-glass or digital thermometer measuring rectal temperature or temperature in other body sites). Other details collected were the type of thermometer, the cut-off used and the method of measuring body temperature; (iv) diagnostic study data (i.e. sensitivity, specificity, TP, TN, FP, FN); (v) mean and standard deviation (SD) of the body temperature measured.

### Quality assessment

The methodological quality of each selected study was assessed according to the Quality Assessment of Diagnostic Accuracy Studies (QUADAS-2) checklist [15] which considers four domains (patient selection, index test, reference standard, and flow and timing), each rated in terms of risk of bias and applicability to the research question. The risk of bias was judged as “low”, “high”, or “unclear”. Each domain included different signalling questions guiding the risk of bias assessment. If all signalling questions received a favorable answer, then the risk of bias was judged as “low”. Concerning applicability, the authors recorded the information on why the study may not have matched the review question. Concerns regarding applicability were rated as “low”, “high”, or “unclear”. At any review stage, disagreements were resolved by discussion or by the involvement of a third investigator.

### Data analysis

For each study, we constructed two-by-two tables and pooled TP, FP, TN, and FN to create separate forest plots to examine the accuracy of different devices to diagnose fever. We used mixed models to fit bivariate meta-analyses, which model sensitivity and specificity while accounting for their correlation [16]. For this purpose, we pooled data at a 38 °C threshold and adopted rectal temperature detected using mercury-in-glass or digital thermometry as the reference standard. We performed a meta-analysis if data were available from at least five studies.

As reported above, for our secondary objective we used NMA methods to use direct and indirect evidence and compare the mean difference of each device using the rectal mercury thermometer as the reference technique. We generated standard errors (SEs) from SDs of the differences or from p-values as appropriate; then we used available between-test correlation coefficients, or their median, to compute adjusted SEs that could not be obtained by conversion of published SDs [17].

We considered the 95% coefficient of reproducibility as a measure of reliability between two tests (i.e. different thermometers) with measurements obtained on the same person [14]. The coefficient of reproducibility is defined as the mean difference (MD) ± 1.96 SD of differences (SDD). In our study, the mean difference is the fixed bias and was estimated using NMA techniques. Once the fixed bias is taken into account, 1.96 × SDDs inform on the random error measurement component. However, meta-analytic methods to estimate pooled SDDs have not yet been developed to the best of our knowledge. Therefore, we presented 1.96 × SDDs for each direct comparison and reported on their variation and the median value for each comparison.

The software STATA 15.2 (StataCorp, 2011; Stata Statistical Software: Release 15. College Station, TX) was used for all analyses. In particular, the ‘network’ suite of commands was used to fit NMAs [18].

### Evidence profile

We evaluated the evidence using the GRADE approach and produced a’Summary of findings’ table for studies that assessed the accuracy of tympanic infrared and temporal artery thermometers to diagnose fever. Studies were initially considered of high quality but were downgraded according to their risk of bias, the directness of evidence (generalizability), consistency, and precision of results across all trials that measured a given specific outcome. Directness refers to the extent to which trial participants, interventions, and outcome measures considered in the included trials are relevant to the review question. Consistency concerns the degree of homogeneity (direction and magnitude) of results across the different studies. Precision describes the grade of uncertainty around the effect estimate, in other words, the width of estimated CIs [19].

We used the STATA *metandi* package [20] to fit bivariate models, the STATA *network* routine to perform NMA [21] and the STATA *metan* function to obtain pairwise meta-analyses [22].

The funder of the study had no role in study design, data collection, data analysis, data interpretation, or writing of the report. The corresponding author had full access to all the data in the study and had final responsibility for the decision to submit for publication.

## Results

### Studies identification and selection

**Fig. 1:**
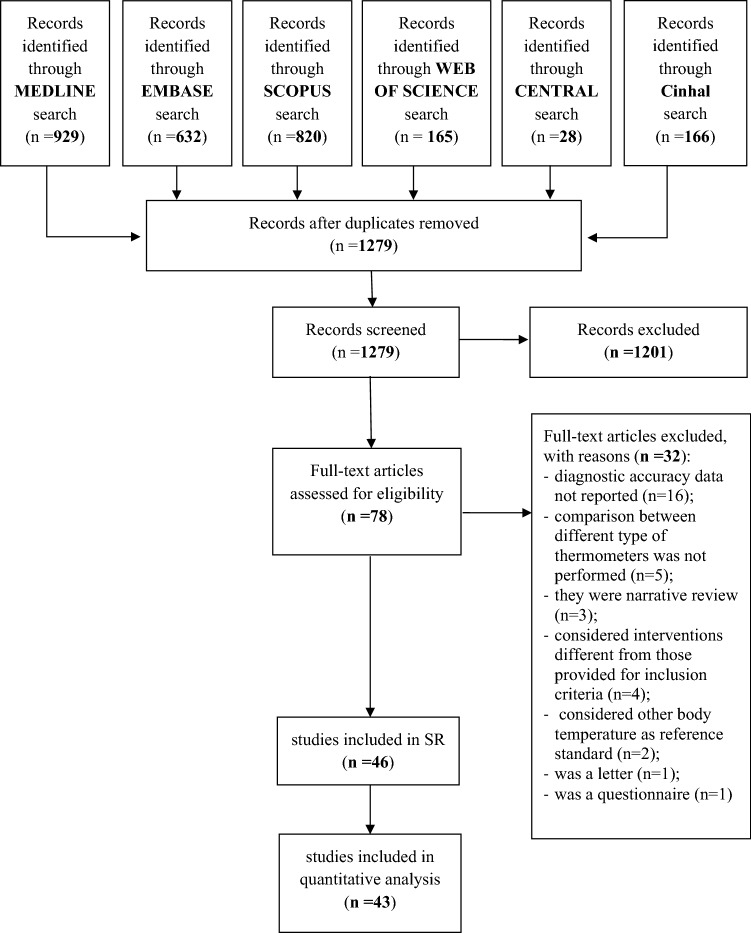
PRISMA flow diagram

The literature search on Medline, Embase, Web of Science (WOS), Scopus, CENTRAL, and Cinhal, after the exclusion of duplicates and irrelevant records, identified 1279 references. Of these, 1201 were excluded because they did not meet the inclusion criteria. Seventy-eight studies were considered eligible for inclusion and their full texts were evaluated for details. Of these, 32 were excluded because (i) diagnostic accuracy data were not reported (*n* = 16); (ii) comparison between different types of thermometers was not performed (*n* = 5); (iii) they were narrative reviews (*n* = 3); (iv) considered interventions different from those provided as inclusion criteria (*n* = 4); (v) considered other body temperature sites as a reference standard (i.e. bladder temperature) (*n* = 2); were a letter (*n* = 1); were a questionnaire (*n* = 1). Finally, a total of 46 studies [23–68] were included in this systematic review (Fig. 1).

### Study characteristics

We included 46 studies (12,602 patients), of whom 32 studies (8321 patients, 66%) enrolled only children, 11 enrolled only adults (1856 patients, 15%) and three studies (2425 patients, 19%) enrolled both adults and children. Nineteen studies (4391 patients) enrolled patients admitted to ED. We included 30 cohort studies, 12 cross-sectional studies, and four randomized controlled trials. Table 1 reports details of the studies included. The number of participants ranged from 15 to 2000. The selected studies were published between 1991 and 2019. We included 43 studies in quantitative analyses, three studies [25, 27, 37] did not provide data allowing the DTA analysis and NMA analysis. Six studies [24, 41, 45, 47, 55, 66] reporting results on measurements were excluded from DTA analyses because the unit of analysis was patients, but was included in the NMA because the unit of analysis was means. For one of them [47], however, we calculated a 2 × 2 table using reported estimates on measurements and prevalence of fever in these patients.

**Table 1:** Characteristics of the individual included studies

Author	Study design	Setting	Population	*No*. patients enrolled	Reference standard (body temperature, device)	Index test (body temperature, device)
Allegaert 2014 [23]	RCT	ED	Children	294	Rectal, digital	Tympanic, infrared Forehead, infrared Temporal artery, infrared
Apa 2013 [24]	Cohort	Pediatric unit	Children	50	Axillary, digital	Tympanic, infrared Forehead, infrared
Balla 2019 [25]	Cross-sectional	Neurological and infection wards	Adult	15	Rectal, digital	Axillary, thermistor
Batra 2013 [26]	Cohort	Pediatric ED	Children	100	Rectal, mercury-in-glass	Axillary, digital Tympanic, infrared Temporal artery, infrared
Berksoy 2018 [27]	Cohort	Pediatric ED	Children	319	Axillary, digital	Forehead, infrared
Brennan 1995 [28]	Cohort	ED	Children	370	Rectal, digital	Tympanic, infrared
Brosinski 2018 [29]	Cohort	ED	Adult and children	251	Rectal, digital	Temporal artery, infrared
Chiappini 2011 [30]	Cross sectional	Pediatric ED	Children	252	Axillary, mercury-in-glass	Forehead, infrared
Dakappa 2016 [31]	RCT	Hospital	Adult	55	Axillary, mercury-in-glass	Tympanic, infrared
Devrim 2007 [32]	Cross sectional	Pediatric hospital	Children	102	Axillary, mercury-in-glass	Tympanic, infrared
Duru 2012 [33]	Cohort	Pediatric unit and ED	Children	300	Rectal, mercury-in-glass	Tympanic, infrared
Edelu 2011 [34]	Cohort	Hospital	Children	800	Rectal, mercury-in-glass	Tympanic, infrared
Forrest 2017 [35]	Cohort	ED	Children	85	Rectal, digital	Axillary, digital Temporal artery, infrared
Gasim 2013 [36]	Cross sectional	ED	Adult and children	174	Axillary, mercury-in-glass	Tympanic, infrared
Goswami 2017 [37]	Cohort	Pediatric unit	Children	210	Rectal, digital	Axillary, digital Temporal artery, infrared
Greenes 2001 [38]	Cross-sectional	ED	Children	304	Rectal, digital	Tympanic, infrared Temporal artery, infrared
Hamilton 2013 [39]	Cohort	ED	Children	205	Rectal or oral, digital	Tympanic, infrared Temporal artery, infrared
Hay 2004 [40]	Cohort	Primary care	Children	94	Axillary, mercury-in-glass	Tympanic, infrared
Hebbar 2005 [41]	Cohort	Pediatric unit	Children	44	Rectal, digital	Axillary, digital Temporal artery, infrared
Isler 2014 [42]	Cohort	Pediatric ED	Children	218	Temporal artery, infrared	Axillary, mercury-in-glass Axillary, digital
Jean Mary 2002 [43]	Cohort	Hospital	Children	198	Rectal, digital	Axillary, infrared Tympanic, infrared
Jensen 2000 [44]	RCT	Surgical unit	Adult	200	Rectal, mercury-in-glass	Tympanic, infrared Oral, digital Axillary, digital Rectal, digital
Kara 2009 [45]	RCT	Pediatric hospital	Children	61	Axillary, mercury-in-glass	Axillary, digital
Kocoglu 2002 [46]	Cohort	Hospital	Children	110	Rectal, mercury-in-glass	Axillary, mercury-in-glass Tympanic, infrared
Leon 2005 [47]	Cross sectional	Intensive care unit	Adult	50	Axillary, mercury-in-glass	Tympanic, infrared
Mogensen 2018 [48]	Cross-sectional	Pediatric department	Children	995	Rectal, digital	Tympanic, infrared Temporal artery, infrared
Mogensen 2018b [49]	Cohort	ED	Adult	599	Rectal, digital	Tympanic, infrared
Morley 1998 [50]	Cohort	Hospital	Children	1090	Axillary, mercury-in-glass	Forehead, infrared
Muma 1991 [51]	Cross sectional	Pediatric ED	Children	224	Rectal, digital	Axillary, digital Tympanic, infrared
Odinaka 2014 [52]	Cohort	Pediatric unit	Children	156	Rectal, mercury-in-glass	Forehead, infrared
Oncel 2013 [53]	Cohort	Maternity unit	Children	120	Rectal, mercury-in-glass	Axillary, digital Forehead, infrared
Paes 2010 [54]	Cohort	Pediatric unit	Children	100	Rectal, digital	Tympanic, infrared Temporal, infrared
Petersen 1997 [55]	Cohort	Neurosurgical unit	Adult	65	Rectal, mercury-in-glass	Tympanic, infrared
Rabbani 2010 [56]	Cohort	Hospital	Adult and children	2000	Oral, mercury-in-glass	Tympanic, infrared
Rajee 2006 [57]	Cohort	ED	Adult	200	Oral, mercury-in-glass	Tympanic, infrared
Schreiber 2013 [58]	Cross-sectional	Pediatric ED	Children	284	Axillary, mercury	Axillary, digital Axillary, galinstan
Schuh 2004 [59]	Cohort	ED	Children	327	Rectal	Forehead
Sehgal 2002 [60]	Cohort	ED	Children	60	Rectal, digital	Tympanic, infrared
Singler 2013 [61]	Cohort	ED	Adult	427	Rectal, digital	Tympanic, infrared Temporal artery, infrared
Smitz 2009 [62]	Cohort	Geriatric unit	Adult	100	Rectal, digital	Tympanic, infrared
Smitz 2000 [63]	Cohort	Geriatric unit	Adult	45	Rectal, mercury-in-glass	Tympanic, infrared
Teller 2014 [64]	Cross-sectional	Private pediatric practice	Children	254	Rectal, digital	Tympanic, infrared Forehead, infrared
Teran 2012 [65]	Cross-sectional	Inpatient unit and ED	Children	434	Rectal, mercury-in-glass	Forehead, infrared Temporal artery, infrared
Van Staaij 2003 [66]	Cohort	Pediatric unit	Children	41	Rectal, digital	Tympanic, infrared
Wilshaw 1999 [67]	Cohort	Clinic	Children	120	Rectal, mercury	Tympanic, infrared Axillary, digital
Yaron 1995 [68]	RCT	ED	Adult	100	Rectal, digital	Tympanic, infrared

### Risk of bias assessment

The results of the methodological quality of the included studies are shown in Appendix 1. The majority of the studies were judged low risk of bias for patient selection and flow and timing. Twenty-six studies (56%) enrolled consecutive or a random sample of patients. Patient enrolment was unclear in 17 studies. The index test domain was judged as unclear in four studies and at high risk in seven studies. Assessors deemed blinding was adequate only in seven studies, and five studies were not blinded regarding the results of the index test and reference standard, but this aspect seemed not to influence the applicability of the study results. In all studies except one, all patients received the same reference standard. Concerns regarding applicability were low for most of the evaluated studies.

###  Diagnostic accuracy estimates

Twenty-eight studies [23, 26, 28–30, 33–35, 38–40, 43, 47–52, 54, 56–59, 62–65, 68] provided data which permitted the extraction of sensitivity and specificity in 10,207 participants, of whom 2729 (27%) had fever according to the reference standard used. The reference standard was a mercury-in-glass or digital thermometer at the rectal site in 19 studies, mercury-in-glass at the axillary or oral sites (seven studies), the digital thermometer at oral/rectal site (one study), or rectal sites with no information on the device (one study). Fifteen studies out of 19 used 38 °C as the cut-off value of temperature for reference devices, four studies used lower values, two studies higher values.

In order to make our results transferrable, we included only studies using a cut-off of 38 °C and a reference standard verification at the rectal site, whether using a digital or a mercury-in-glass thermometer.

**Fig. 2:**
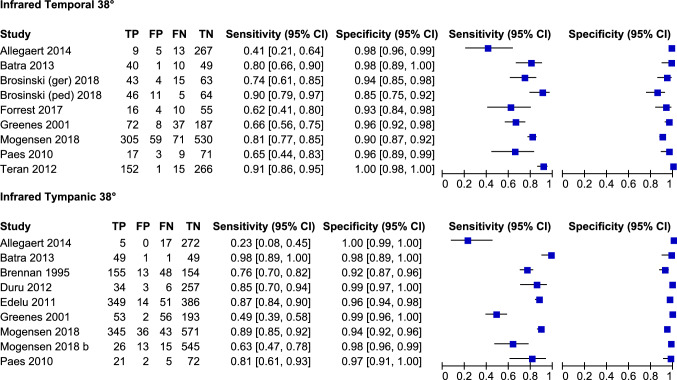
Forest plots of the accuracy of infrared thermometer at the temporal and the tympanic sites (index devices) versus mercury-in-glass or digital thermometer at the rectal site (reference standard device) among studies using 38 °C as cut-off values of temperature for index and reference standard devices. *TP* true positive, *FP* false positive, *FN* false negative, *TN* true negative, *CI* confidence interval

In 9 studies (2533 participants, 885 with fever) using temporal artery infrared thermometry at a threshold of 38 °C, sensitivity varied between 0.41 and 0.91, while a high specificity (from 0.85 to 1.00) was achieved (Fig. 2). The meta-analytic estimates were 0.76 (95% CI 0.65, 0.84) for sensitivity and 0.96 (0.92, 0.98) for specificity (Table 2). This means that adopting a 38 °C index test threshold, there are very few false positives, even at the relatively high prevalence of fever at 30%, but there are several false negatives, so the test is useful to rule in the disease when positive. The certainty of the evidence, after downgrading by one level for risk of bias, was low for patients with fever due to imprecision of sensitivity estimates, and moderate for patients without fever (Table 2).

**Table 2:** Summary of findings tables

DTA (Mercury-in-glass or digital rectal at 38 °C as cut-off as reference device)						NMA		
Device		No. of studies (no. of patients)	Estimate (95% CI)	Effect per 1000 patients tested	Certainty of evidence	No. of direct studies ( no. of patients)	Mean difference (95% CI)	Coefficient of reproducibility, median (range)
Infrared temporal	TP	9 (885)	Sensitivity: 0.76 (0.65, 0.84)	228 (180–252)	⨁⨁◯◯ Low	1 (434) vs. Mercury-in-glass rectal	− 0.09 (− 0.42, –0.24)	0.53 (0.53, 0.53)
	FN			72 (48–120)				
	TN	9 (1648)	Specificity: 0.96 (0.92, 0.98)	672 (644–686)	⨁⨁⨁◯ Moderate	8 (2206) vs. Digital rectal	− 0.09 (− 0.33, –0.16)	1.2 (0.81, 1.5)
	FP			28 (14–56)				
Infrared tympanic	TP	9 (1,279)	Sensitivity: 0.77 (0.60, 0.88)	231 (180–264)	⨁⨁◯◯ Low	6 (840) vs. Mercury-in-glass rectal	− 0.22 (− 0.49, –0.04)	0.73 (0.56, 1.9)
	FN			69 (36–120)				
	TN	9 (2583)	Specificity: 0.98 (0.95, 0.99)	686 (665–693)	⨁⨁⨁◯ Moderate	12 (3520) vs. Digital rectal	− 0.22 (− 0.43, − 0.01)	1.1 (0.24, 1.5)
	FP			14 (7–35)				

Similarly, in nine studies (3862 participants, 1279 with fever) using tympanic infrared thermometry at a threshold of 38 °C, high specificity was achieved (from 0.92 to 1.00); however, sensitivity varied between 0.49 and 0.98 in eight studies and was 0.23 in the study with perfect specificity (Fig. 2). The meta-analytic estimates were 0.77 (0.60, 0.88) for sensitivity and 0.98 (0.95, 0.99) for specificity (Table 2). After downgrading by one level for risk of bias, the certainty of the evidence was low for patients with fever due to imprecision of sensitivity estimates, and moderate for patients without fever.

No statistically significant difference was found between sensitivity and specificity estimates with infrared tympanic vs. temporal artery thermometry, which is unsurprising given the high heterogeneity in sensitivity.

There were three or fewer studies on other devices/sites and cut-offs (Appendix 2), thus meta-analyses were not possible.

### Mean differences between thermometers using network meta-analyses

Thirty-six studies [24, 28–33, 35, 36, 38, 40–56, 58, 60–63, 65–68] provided data that permitted the extraction of temperature means in 9,878 participants.

Twenty-one studies included in this analysis were incompletely reported regarding the correlation between measurements on the same person. Specifically, four studies reported the SD of the differences between each pair of device-site, 10 studies reported p-values of paired tests, and seven studies reported correlation coefficients, with one study reporting two parameters and the other studies reporting no data to extract the within-subject correlation. We adopted the strategy reported in the Methods to overcome this issue.

Appendix 3 presents a network map. There was no sign of overall (*p* = 0.9795) or loop-specific between-study heterogeneity, possibly also due to the precision of within-study estimates (small SDs) as compared to the between-study SD (tau) which was 0.397 °C, meaning that the additional uncertainty due to heterogeneity was almost + − 0.8 °C in any 95% predictive interval.

Appendix 4 shows all studies in direct meta-analyses (boxes with horizontal bars) together with the NMA estimate (diamonds). The direct comparison between axillary mercury and infrared tympanic thermometry was very heterogeneous in 6 studies, with extreme values of mean differences ranging from less than − 1 °C to + 1 °C. On the contrary, infrared tympanic thermometry, compared to rectal mercury-in-glass (five studies) or rectal digital thermometry (eight studies), showed consistent differences, suggesting less variation in their results.

**Fig. 3:**
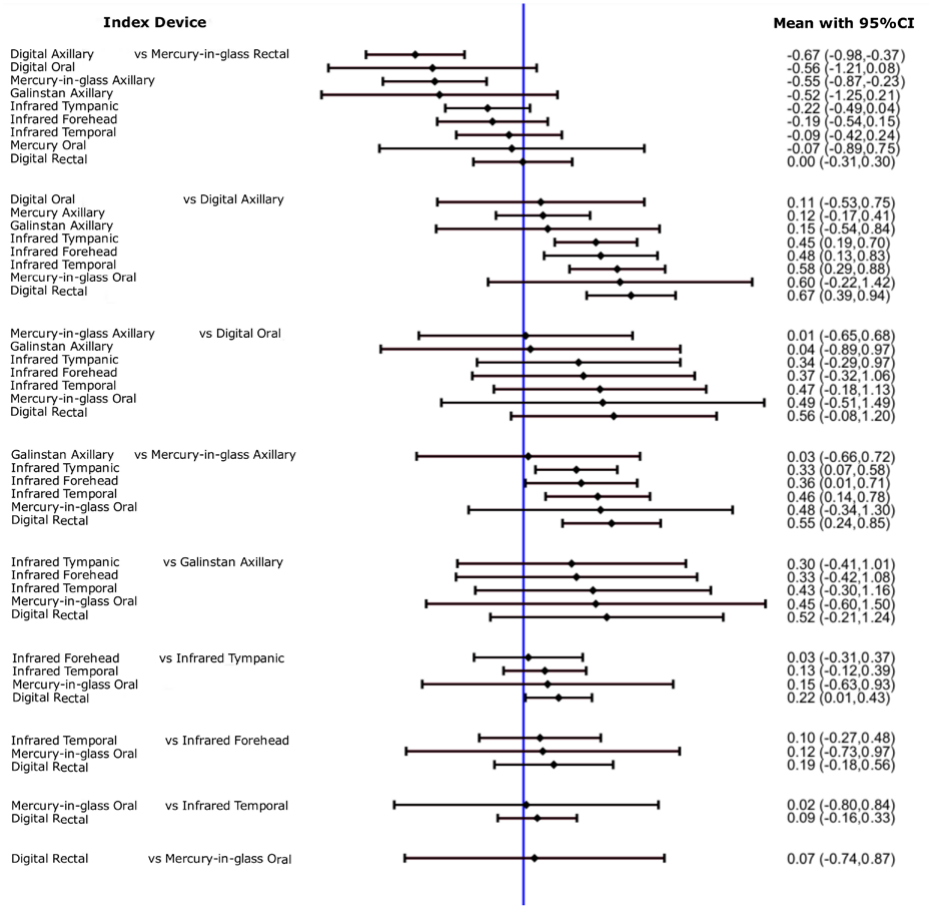
Forest plot of mean differences among thermometers at different anatomical sites from network meta-analysis

Figure 3 presents all pairwise mixed estimates with 95% CIs. Assuming rectal mercury-in-glass thermometry as the reference, axillary digital thermometry was significantly lower by − 0.67 °C (− 0.98, − 0.37), as was also axillary mercury-in-glass thermometry (− 0.55 °C [− 0.87, − 0.23]); a similar difference was obtained for oral digital, and axillary galinstan thermometry, but with greater imprecision crossing significance (− 0.56 °C [− 1.21, 0.08] and − 0.52 °C [− 1.25, 0.21], respectively). All other differences were also in the direction of a lower temperature with respect to rectal thermometry by − 0.22 °C to 0.00 °C, but none was statistically significant. All other pairwise differences among devices were small in most cases but imprecisely estimated.

### Description of random error for each direct comparison

**Fig. 4:**
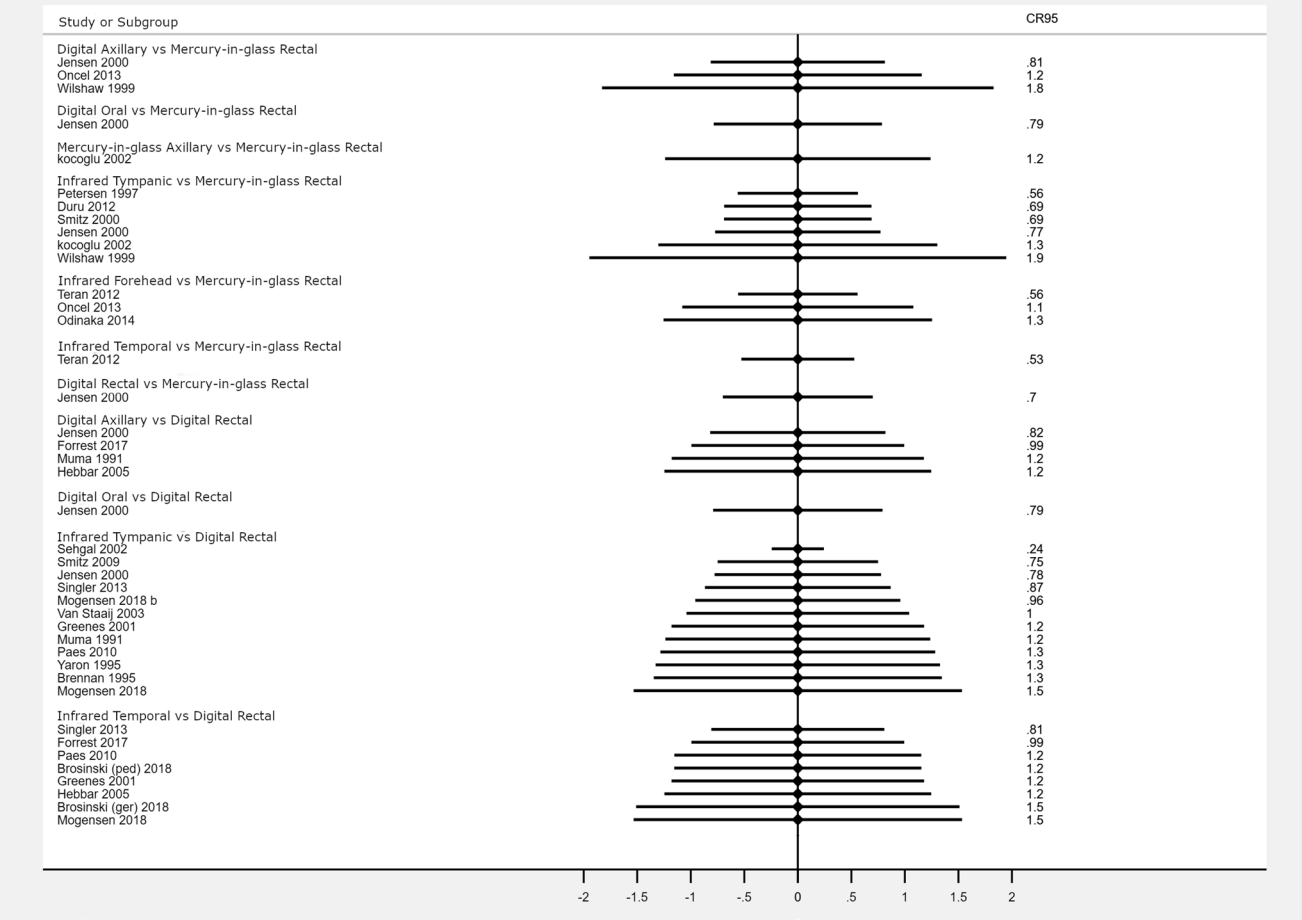
Forest plot of 95% coefficient of reproducibility (95% CR) of thermometers at different anatomical sites from the meta-analysis

Figure 4 presents the 95% coefficient of reproducibility (95% CR). The mean 95% CR value of 73 direct comparisons between devices was 1.06 °C, with 19 comparisons below 0.82 °C, 17 between 0.82 and 1.08 °C, 19 between 1.08 and 1.24 °C, and 18 exceeding 1.24 °C. The median 95%CR vs. rectal mercury-in-glass thermometry was 1.16 °C for axillary digital thermometry (three studies), 0.79 °C for digital oral thermometry (one study), 0.70 °C for digital rectal thermometry (one study), 0.73 °C for tympanic infrared thermometry (six studies), 1.08 °C for infrared forehead thermometry (three studies) and 0.53 °C for infrared temporal thermometry (one study).

### Sensitivity analyses

We restricted the NMA to 24 studies conducted on children and found a similar pattern of differences, although they were less precise due to the reduced size. There were too few studies to estimate accuracy in a specific setting, such as ED.

## Discussion

This systematic review summarizes published data from 46 studies evaluating different types of thermometers to measure body temperature. The gold standard to measure core temperature is the rectal temperature as it better reflects a true central temperature. However, it has several drawbacks including impracticability, discomfort, and, although rare, possible complications, such as perforation or transmission of microorganisms.

Our meta-analysis showed that alternative peripheral thermometers were not always accurate at estimating central core temperature, with a tendency to underestimate it up to one degree Celsius. Another challenge is the pervasive presence of a random error that afflicts all thermometers and that can be estimated to add an extra degree of error. The interplay between the fixed and random error originating by the use of different thermometers might generate, in the worst case, clinically relevant differences in the order of two degrees Celsius. The uncertainty associated with thermometers and the resulting implications for decision-making led researchers to use a relatively high fever threshold of 38 °C for both the index and the reference test. With this value, the specificity of peripheral thermometers is high and adequate to confirm fever when detected, but the sensitivity is much lower, making it difficult to exclude fever for temperatures below 38 °C.

Our network meta-analysis showed that axillary temperature, determined with both mercury and digital thermometers, was significantly lower by − 0.65 °C and − 0.67 °C, respectively, than body temperature measured with rectal mercury-in-glass thermometry, while infrared (tympanic, temporal artery, forehead) devices were slightly better estimators of body temperature, showing smaller, non-significant differences. It is to be noted that the mean difference of the rectal temperatures measured with mercury-in-glass or digital thermometers is nil with mild variability and this makes the choice of these two devices as mixed reference standard reasonable. When the aim is to diagnose a febrile state, both in children and adults, the accuracy estimates of both infrared tympanic and temporal thermometry are the best in our review, and they are supported by the largest body of evidence. Previous reviews, despite the variability in the methodology used and in the included studies, also concluded that tympanic and temporal artery thermometers are more accurate, achieving high specificity but insufficient sensitivity when assessed against rectal thermometry [2, 69, 70]. Some of these reviews also conducted meta-analyses of the mean difference between peripheral and rectal thermometry and found that the mean difference was about 0.2 °C [69]. Niven et al. calculated 95% coefficients of reproducibility as twice the SDs but did not explain how SDs were pooled across studies [2].

Rectal temperature is just a proxy of the real (and latent) body temperature. For instance, if the reference device tended to overestimate the real temperature, the “real” sensitivities of the index devices could be possibly higher than the ones illustrated in the paper, because some of the reported false negatives would in fact be true negatives.

According to the GRADE evaluation, the overall certainty of our estimates was moderate, due to some limitations in the design of several studies, or considerable heterogeneity across studies. Heterogeneity could be due to several reasons: measuring temperature in different body sites; concomitant inclusion of children and adults; a threshold effect caused by the use of different offsets by manufacturers to obtain adjusted temperatures according to thermometer technology; and intra- and inter-operator variability of measurements. The risk of bias assessment showed that the study populations were in general selected with convenience samples of participants. Blinding was almost nonexistent, but we considered this as non-fundamental since most technologies give a digital result that has to be recorded without interpretation. The timing between the index and reference methods was usually reported. Finally, there was great heterogeneity among included studies which reduced the quality of evidence.

We suggest that in future studies temperatures should be measured independently at specific sites in a consecutive series of eligible individuals. All thermometers should be previously calibrated. Details on placement time, patient stabilization, and mode of use of thermometers should be provided. Temperature readings should be carried out concurrently or sequentially and the time between measurements clearly documented. However, body temperature should be evaluated in relation to individual variability, since it varies with respect to age, gender, site of measurement, type of thermometer and presence of disease.

As shown above, we found a cut-off of 38 °C was highly specific, but not sensitive enough to detect fever with an equivalent rectal cut-off so that thermometry could be used to exclude or rule out fever. If the aim of body temperature measurement is to triage subjects with high sensitivity (confirm or rule in fever, SpIN approach), future research should use an external body site cut-off of about 37 °C to confirm a rectal temperature exceeding 38 °C. We highlight that the balance of sensitivity and specificity should not be assumed to be stable when the cut-off is changed on the basis of our data, since ROC curves are often asymmetric and the overall accuracy (e.g. DOR) at high specificity may not match the value found at high sensitivity.

Fever is one of the most common patient complaints and signs in emergency departments and is often caused by infection. Other sources include pulmonary embolism, intracranial hemorrhage, medication, or malignancy. Determining a fever represents a fundamental step of health status assessment, with a bearing on medical decisions; for instance, fever can contribute to the empirical assessment of bacterial infections, leading to the prescription of antibiotics. The presence of fever might lead to quarantine in patients suspected of Covid-19 infection or admission to the hospital. Temperature measurement is imperfect and requires awareness and appreciation of its limits. Health professionals should consider that large errors are found when measuring temperature. Therefore, they should complement temperature with additional clinical elements (e.g. medical history, heart rate, and palpitations). Health professionals should adopt quality assurance procedures for fever diagnosis in order to limit variation in clinical practice, enhancing the education on thermometer use and measurement interpretation, similarly to what has been done with the promotion of hand hygiene practice. A simple approach to decrease random error would be to increase the number of measurements, an action that should be considered when the temperature has strong decision-making implications.

When a temperature cut-off of 38 °C is used to define fever, several peripheral thermometers proved to be specific, but not sensitive when rectal thermometry is used as a reference standard, meaning that finding a temperature below 38 °C does not rule out fever. Among all devices, infrared tympanic and temporal thermometers were better estimators of central temperature and achieved consistent performances across studies. Most thermometers are afflicted with substantial random error. The under-appreciation of the uncertainty in measuring temperature while practicing medicine might have serious consequences: the limited accuracy and reproducibility of thermometers may translate into weak decision-making, a huge waste of resources, and suboptimal patient and population health outcomes.

## Electronic supplementary material

Below is the link to the electronic supplementary material.

Supplementary file1 (DOCX 799 kb)
