# Supplementary material for: The diagnostic accuracy of digital, infrared and mercury-in-glass thermometers in measuring body temperature: a systematic review and network meta-analysis
Source: Intern Emerg Med. 2020 Nov 25;16(4):1071–83. doi: 10.1007/s11739-020-02556-0 (PMC7686821; doi:10.1007/s11739-020-02556-0)
Supplement: Supplementary file 1 — Supplementary file1 (DOCX 799 kb) [file 11739_2020_2556_MOESM1_ESM.docx]

**Title**

The diagnostic accuracy of digital, infrared and mercury-in-glass thermometers in measuring body temperature: a systematic review and network meta-analysis

**Authors**

1. Valentina Pecoraro^1^, Davide Petri^2^, Giorgio Costantino^3^, Alessandro Squizzato^4^, Lorenzo Moja^5^, Gianni Virgili^6^, Ersilia Lucenteforte^2^

**Author affiliations**

^1^ Department of Laboratory Medicine and Pathology, Ospedale Civile Sant’Agostino Estense, AUSL Modena, Modena, Italy

^2^ Department of Clinical and Experimental Medicine, University of Pisa, Pisa, Italy

^3^ IRCCS Fondazione Ca’ Granda, Ospedale Maggiore Policlinico, UOC pronto Soccorso e Medicina D’Urgenza, Università degli Studi di Milano, Milan, Italy

^4^ Department of Medicine and Surgery, University of Insubria, Como, Italy

^5^ Department of Biomedical Sciences for Health, University of Milan, Milan, Italy

^6^ Department of Neurosciences, Psychology, Drug Research and Child Health (NEUROFARBA), AOU Careggi, Florence, Italy

**Corresponding author**

Ersilia Lucenteforte, ScD, PhD

Department of Clinical and Experimental Medicine, University of Pisa,

Via Roma, 10 - 56126 Pisa, Italy.

E-mail: ersilia.lucenteforte@unipi.it

**List of Appendices**

[**Appendix 1.** Quality of the individual included studies 2](#_Toc54166897)

[**Appendix 2.** Forest plots with accuracy of several index devices versus mercury-in-glass or digital thermometer at the rectal site using 38° C as cut-off values. 3](#_Toc54166898)

[**Appendix 3.** Network map of examined diagnostic thermometers and the anatomical region where the body temperature measurement was made. 4](#_Toc54166899)

[**Appendix 4.** Direct meta-analyses effects of examined individual diagnostic thermometer and the anatomical region. 5](#_Toc54166900)

# **Appendix 1.** Quality of the individual included studies


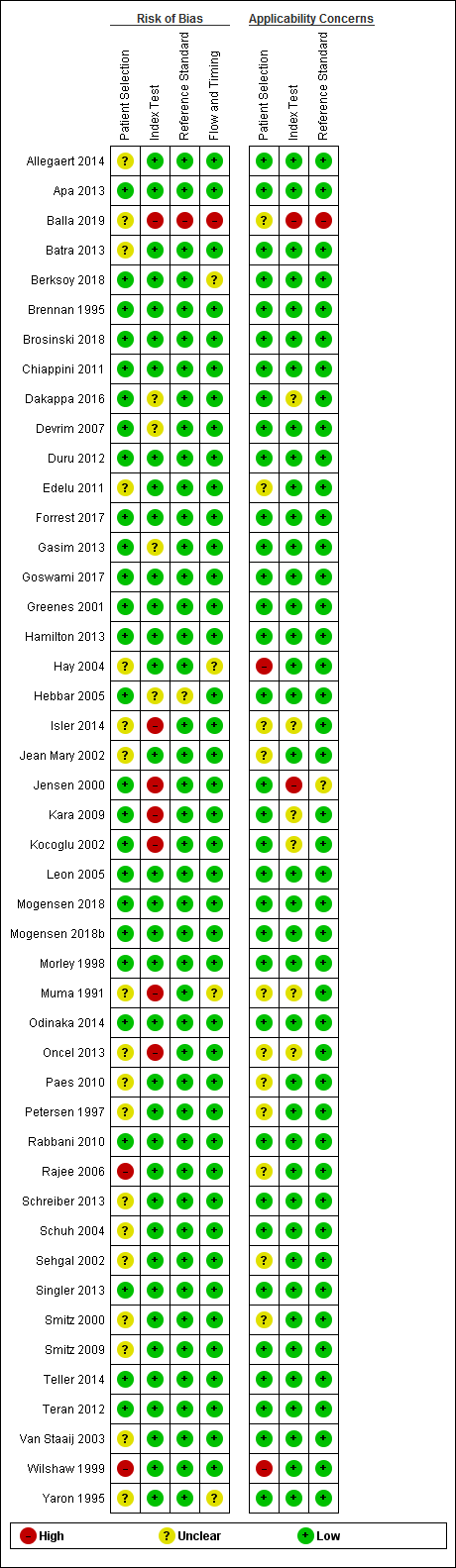


# **Appendix 2.** Forest plots with accuracy of several index devices versus mercury-in-glass or digital thermometer at the rectal site using 38° C as cut-off values.


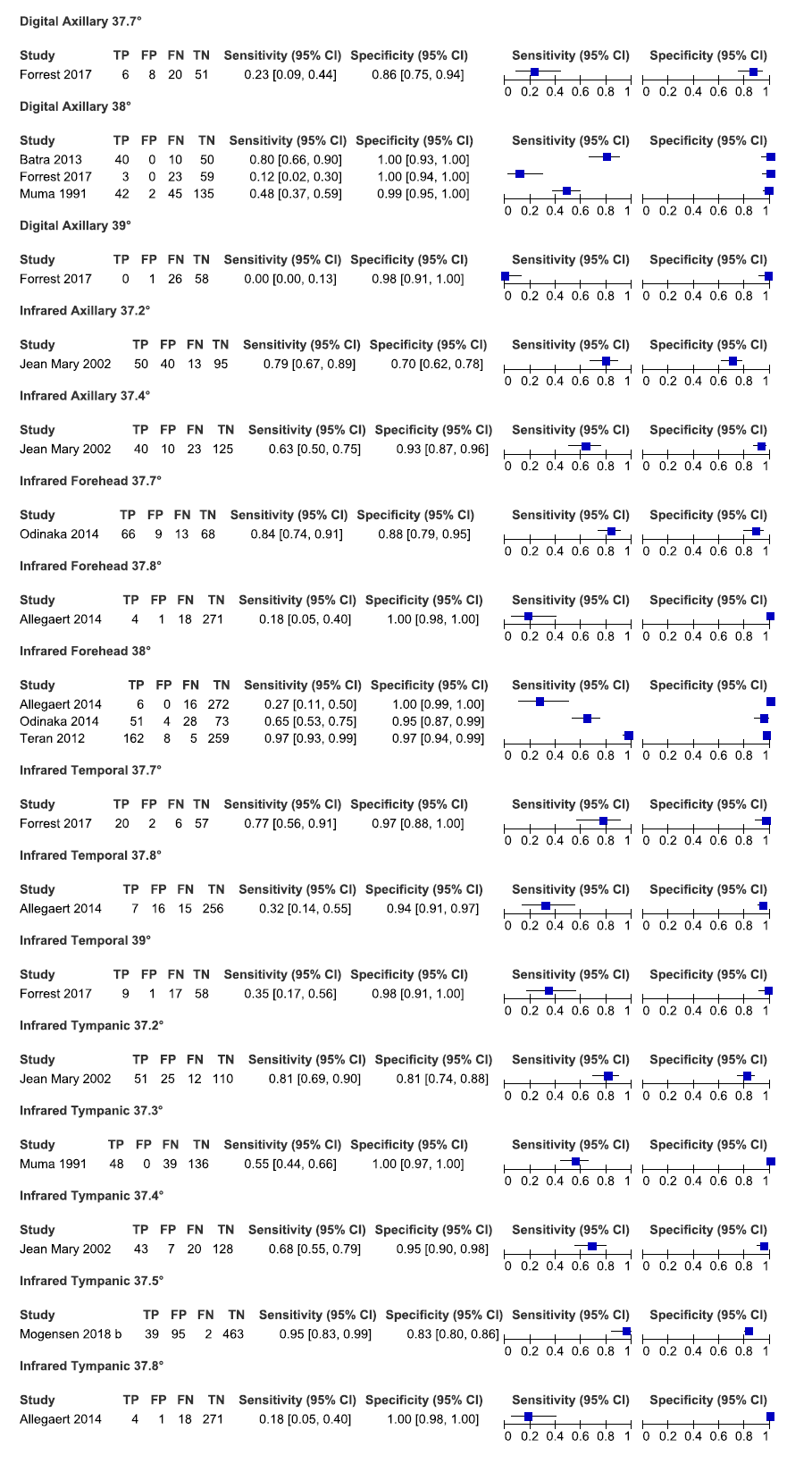
Each comparison provides information on plotted studies, the index test and the anatomical region where the measurement was made, and the temperature cut-off for fever. TP=true positive; FP=false positive; FN=false negative; TN=true negative; CI=confidence interval.

# **Appendix 3.** Network map of examined diagnostic thermometers and the anatomical region where the body temperature measurement was made.

Network plot considers digital thermometer at the rectal, the oral, and the axillary sites, mercury-in-glass thermometer at the rectal, the oral, and the axillary sites, infrared thermometer at the tympanic, the temporal, and the forehead sites, and galinstan thermometer at the axillary site.


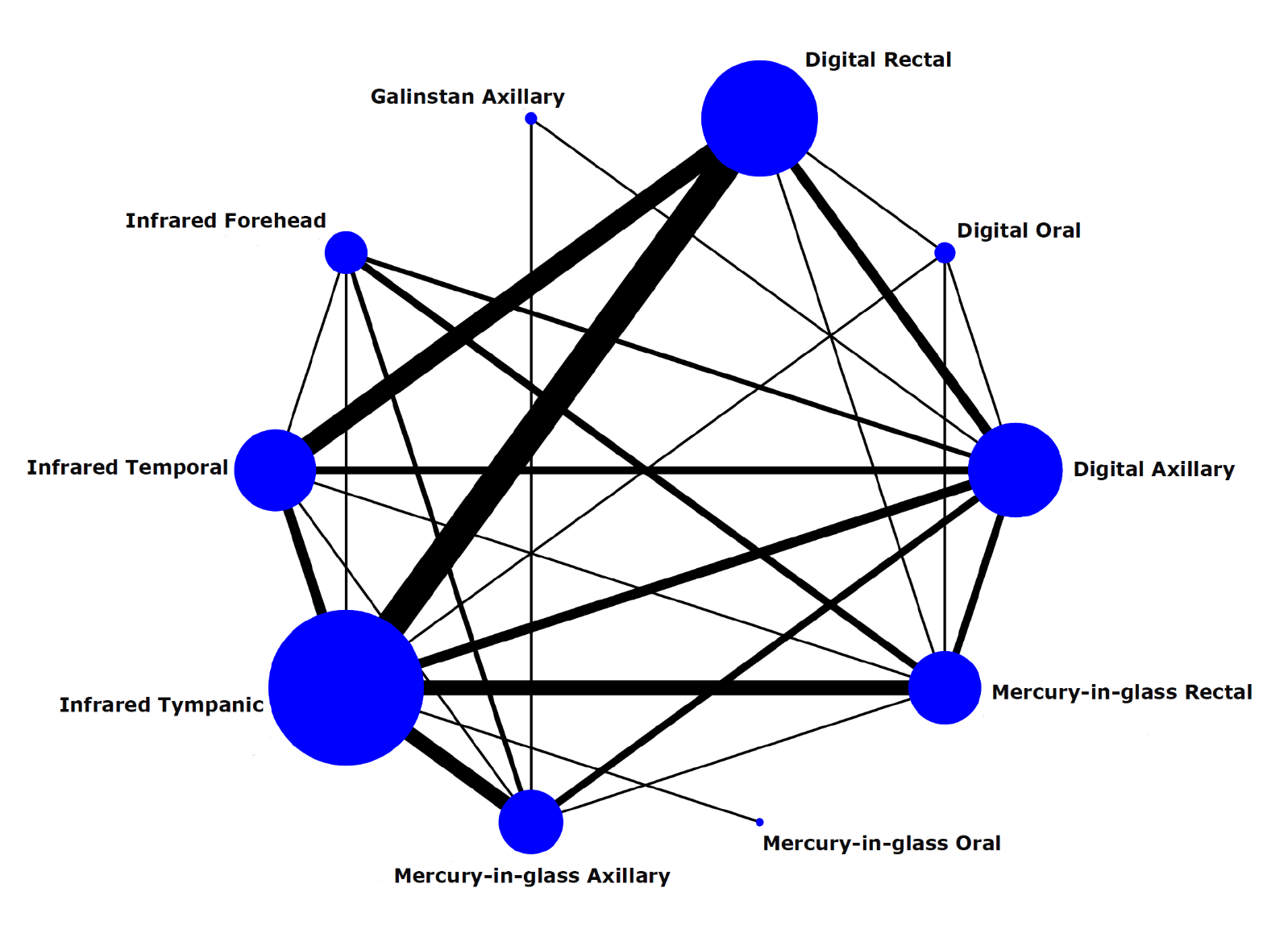


#
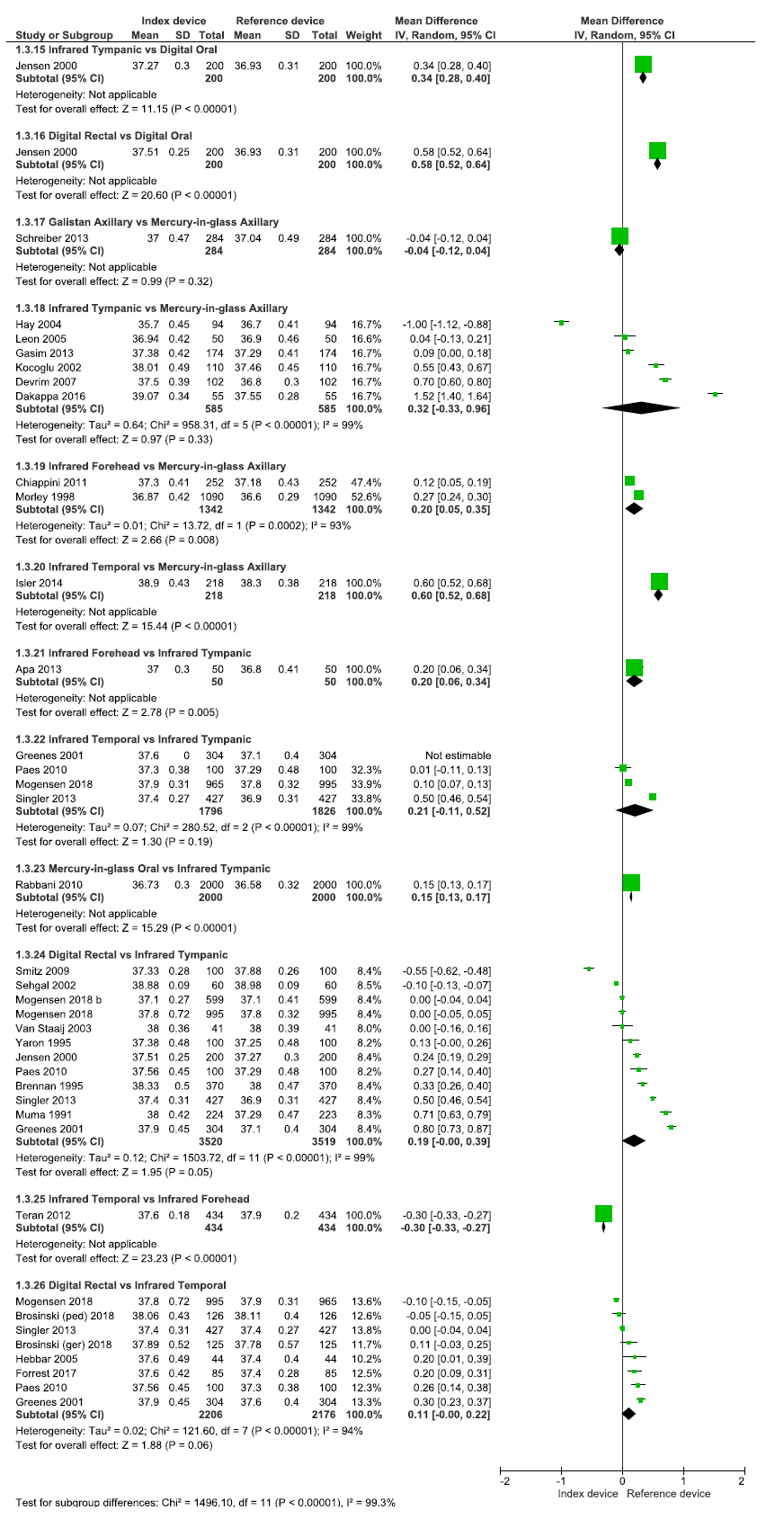
**Appendix 4.** Direct meta-analyses effects of examined individual diagnostic thermometer and the anatomical region.
